# Model Organisms to Study Autophagy

**DOI:** 10.3390/cells12182212

**Published:** 2023-09-05

**Authors:** Qiuhong Xiong, Ludwig Eichinger

**Affiliations:** 1 Shanxi Provincial Key Laboratory of Medical Molecular Cell Biology, Key Laboratory of Chemical Biology and Molecular Engineering of Ministry of Education, Institutes of Biomedical Sciences, Shanxi University, No. 92 Wucheng Road, Taiyuan 030006, China; 2 Center for Biochemistry, Medical Faculty, University of Cologne, Joseph-Stelzmann-Str. 52, 50931 Cologne, Germany

**Keywords:** autophagy, model organism, function, non-canonical autophagy

## Abstract

Autophagy is the major lysosomal pathway for the clearance of proteins, organelles and microbes in eukaryotic cells. Therefore, autophagic dysfunction can lead to numerous human diseases, like cancer or neurodegeneration, and may facilitate infections by pathogens. However, despite tremendous advances in the understanding of autophagy over the past decades, the functions and regulations of autophagy-related proteins in canonical and non-canonical autophagy are still not fully resolved. The Special Issue “Model Organisms to Study Autophagy” organized by *Cells* includes six original articles and one review that show the latest achievements in autophagy research using different model organisms. The Special Issue summarizes and discusses different aspects of autophagy that open new avenues in understanding autophagy functions and mechanisms.

The first description of autophagy genes (*Atg*) was in the yeast *Saccharomyces cerevisiae* in 1993 by the Nobel laureate Yoshinori Ohsumi [1]. In the coming years, 15 *Atg* genes were identified through genetic screens by his group and, independently, by several other research groups [2,3,4,5,6,7]. To date, more than 40 *Atg* genes have been identified through studies in different organisms [8]. Research with higher eukaryotes revealed that these *Atg* genes are highly conserved, demonstrating the impressive value of model organisms for autophagy research [9,10,11]. Further research showed that, apart from their functions in canonical autophagy, many autophagy genes in unicellular and multicellular eukaryotes have additional roles in non-canonical autophagy, e.g., in secretion, endocytosis or cytokinesis [12]. To further our understanding of autophagy functions in unicellular and multicellular organisms, *Cells* organized the Special Issue entitled “Model Organisms to Study Autophagy”, which includes six original articles and one review. In the following part, these manuscripts are briefly summarized.

Originally lipophagy, the selective autophagy of lipid droplets (LDs) was mainly studied in the yeast *Saccharomyces cerevisiae* [13]. To further analyze this mechanism, Kumar et al. developed the yeast *Komagataella phaffii* (formerly *Pichia pastoris*) as a new model for lipophagy [14]. Here, the authors discovered Cue5 as a new stationary phase lipophagy substrate. The Cue5 protein belongs to a protein family called CUET. This family includes Cue5 in yeast and toll-interacting protein (TOLLIP) in humans. The Cue5 and TOLLIP proteins possess at least two common structural features, the ubiquitin-binding CUE (coupling of ubiquitin conjugation to endoplasmic reticulum (ER) degradation) domain and AIM, the Atg8-interacting motif. The authors found that the accumulation of Cue5 on LDs and its degradation by stationary phase lipophagy strongly depended on the ubiquitin-binding CUE domain and Prl1, the positive regulator of lipophagy 1. However, unlike Prl1, Cue5 was dispensable for stationary phase lipophagy, suggesting that Cue5 is rather a new substrate for this pathway. The authors propose that a similar Prl1-dependent accumulation on LDs as for Cue5 might be employed by Prl1 to recruit another ubiquitin-binding protein that is essential for S-phase lipophagy [15].

*Tetrahymena thermophila* is a free-living ciliate in water ecosystems worldwide [16]. The organism displays nuclear dimorphism containing a germline micronucleus (MIC) and a somatic macronucleus (MAC). *Tetrahymena* is an ideal model for the study of nucleaphagy as during its sexual reproduction, the parental macronucleus (paMAC) is degraded via autophagy (nucleaphagy) with the development of a new macronucleus [17]. In the present study, the authors showed that Atg5 colocalized with the paMAC during the sexual reproduction of *Tetrahymena* and mediated the degradation of the paMAC by promoting the lipidation of Atg8.2. Consequently, Atg5 deficiency impaired the degradation of the paMAC [18].

The social amoeba *Dictyostelium discoideum* is a well-established model organism for the investigation of the autophagic process [9]. In their paper, the authors further analyzed the global proteome profiles of wild-type AX2, ATG9¯, ATG16¯ and ATG9¯/16¯ strains from tandem mass tag (TMT) proteomics data [19] and performed co-immunoprecipitation experiments followed by mass spectrometric analysis of AX2 wild-type and ATG16¯ cells expressing the 20S proteasomal subunit PSMA4 tagged with GFP. Their results suggest that proteaphagy is severely hampered in autophagy-deficient *D. discoideum* mutants. As a consequence, the ratio of less-active or inactive proteasomes increases, causing a dramatic decrease in proteasomal activity and deranged protein homeostasis [20].

Schürmanns et al. use the filamentous ascomycete *Podospora anserina* as a model for the study of cellular homeostasis and aging [21]. Previously, they found that the absence of the sorting nexin PaATG24 resulted in impairments in different types of autophagy and a shortened lifespan [22]. Here, they show that an oleic acid diet leads to longevity of the wild type and the PaAtg24 deletion mutant (ΔPaAtg24). The authors further analyzed the underlying mechanisms and found that the oleic acid diet abrogates the autophagy defect and reduces the generation of reactive oxygen species (ROS) in the *P. anserina* ∆PaAtg24 mutant by altering membrane trafficking [23].

Autophagy and mTOR signaling are important for the maintenance of podocyte homeostasis; however, the mechanistic role of these pathways for the glomerular filtration barrier remains unclear. Spitz et al. used *Drosophila* nephrocytes as an established podocyte model to unravel the role of mTOR-dependent autophagy. The authors found that proper mTOR signaling is essential for nephrocyte cell size, survival and nephrin expression. Furthermore, they discovered a direct mTOR/autophagy-dependent regulation of the slit diaphragm architecture [24].

The retinal degeneration 10 (rd10) mouse model is widely used to study retinitis pigmentosa (RP), an inherited retinal disease resulting in the progressive degeneration of retinal photoreceptors and ultimately leading to blindness. In many forms of RP, the disease is characterized by the degeneration of rods followed by cones [25]. Here, Yamoah et al. found that progressive neurodegeneration in the rd10 mouse retina is associated with early disturbances of proteostasis and autophagy, along with abnormal cytoplasmic RNA binding protein (RBP) aggregation and altered ER-Ca^2+^ homeostasis. These changes occurred before any noticeable photoreceptor degeneration [26]. Their findings may contribute to our understanding of the spread of pathology in many age-related progressive neurodegenerative diseases.

Autophagy is crucial for the specific recognition and elimination of invading pathogens such as bacteria and viruses, and this process was termed xenophagy [27,28]. However, many pathogens deploy highly evolved mechanisms to evade autophagic degradation, and some even exploit autophagy to ensure their survival [28,29]. Thus, the fight against pathogen infections would benefit from a better understanding of the mechanisms of xenophagy. Bębnowska and Niedźwiedzka-Rystwej focused in the first part of their review on the use of *Lagovirus europaeus* as a research model in acute liver failure (ALF) and viral hemorrhagic disease (VHD) [30]. In addition, the authors also summarized the progress on the role of autophagy in viral hepatitis and viral hemorrhagic fevers and the relations impacting its viral pathogenesis [30].

## Conclusions

Much progress has been made in understanding the molecular mechanisms of autophagy; however, the autophagy research field still faces numerous unanswered questions and challenges [31]. For example, many of the results for the interplay between host and pathogen in xenophagy are based mainly on in vitro systems, while results from in vivo research are very limited. Furthermore, recent genetic studies on humans have identified a number of mutations in autophagy genes that are associated with human disease, emphasizing the importance of autophagy for human health [32]. It is very likely that further mutations in autophagy genes involved in the pathogenesis of human diseases await their identification. Finally, we can be certain that model organisms will continue to significantly contribute to further progress in the understanding of the diverse functions of autophagy.
